# Role of Nutrition in Cognitive Development and Academic Performance During Adolescence: A Comprehensive Review

**DOI:** 10.7759/cureus.96189

**Published:** 2025-11-06

**Authors:** Ayesha Nikalansooriya, G.R. Nipuni N Waidyarathna, Lalindra S Kaththiriarachchi, Ananda Chandrasekara

**Affiliations:** 1 Division of Science and Technology Policy Research, National Science Foundation, Colombo, LKA; 2 Department of Pre-clinical Sciences, Faculty of Medicine, General Sir John Kotelawala Defence University, Rathmalana, LKA; 3 Department of Nutrition and Dietetics, Wayamba University of Sri Lanka, Makadura, LKA

**Keywords:** academic performance, adolescence, anthropometric assessments, biochemical markers, cognitive functions, dietary patterns, nutrition interventions

## Abstract

Adolescence is a vital period marked by rapid physical, cognitive, and emotional development. Proper nutrition is crucial to meet increased physiological needs and support cognitive growth and academic performance. This review examines the relationship between adolescent nutrition and cognitive function, emphasizing dietary patterns, anthropometric assessments, and biochemical markers in shaping health and academic outcomes. A comprehensive literature review was conducted to assess the impact of nutritional status on adolescent cognitive function. The review explores nutritional deficiencies, dietary habits, and their neurocognitive effects, focusing on key nutrients such as protein, iron, zinc, and vitamin D. The review also addresses the double burden of malnutrition among Sri Lankan adolescents due to rapid dietary and lifestyle transitions. Nutritional deficiencies, including protein-energy malnutrition and micronutrient inadequacies, have significant effects on cognitive functions, learning, and brain health. Balanced dietary patterns, such as the Mediterranean diet, are associated with improved neurocognitive outcomes, while unhealthy dietary habits may negatively impact cognitive development. The review identifies a knowledge gap regarding the role of nutrition in Sri Lankan adolescents, emphasizing the need for targeted nutrition interventions. The review highlights the need for targeted nutrition interventions and policy measures, with schools serving as critical settings for implementation. Future research should adopt longitudinal designs to establish causal relationships between nutrition and cognition. Addressing nutritional disparities, improving facility access, and integrating nutrition-focused strategies into public health policies could significantly enhance cognitive and academic outcomes for adolescents in low- and middle-income countries.

## Introduction and background

Adolescents, defined as individuals between the ages of 10 and 19 years, constitute 16% of Sri Lanka’s population and are experiencing rapid physical, cognitive, and emotional changes [1]. However, research on this demographic remains fragmented, particularly in low- and middle-income countries (LMICs) like Sri Lanka, where nutritional transitions (e.g., increase of high-energy-density food consumption) coexist with persistent micronutrient deficiencies [2]. While adolescence is often regarded as a healthy life stage, emerging evidence suggests that poor dietary habits during this period may have long-term consequences for cognitive function and academic achievement [3], a link underexplored in Sri Lanka [1]. Adolescence is a critical developmental transition between childhood and adulthood, characterized by significant physical growth, physiological maturation, and psychological transformation [2]. This period brings profound changes in social cognition and relationships and develops greater independence. Although they are among the healthiest, this life stage presents unique nutritional demands and cognitive challenges that can significantly impact both immediate development and long-term health outcomes".

Importance of nutrition during adolescence

Nutrition is one of the most basic needs of life, necessary for day-to-day living and health. Its adaptability is very much dependent on how much food and what kind of food one eats, i.e., whether an individual is getting enough food to be healthy [3]. Nutrition from this perspective is the quality of calories and nutrients within the diet available to the individual [4]. Adequate caloric and nutrient intake is even more crucial during adolescence, not only to maintain health but also to promote physical and cognitive development during a time of rapid growth and development [5].

These habits nowadays have evolved to a higher consumption of calorically dense and nutrient-poor fast foods, and the convenience and availability of fast foods frequently trump their quality. Consequently, this phenomenon has led to many adolescents ignoring nutrient-dense food choices like vegetables and fruits [6]. These patterns of diet pose even greater problems, given that adolescence is a stage marked by increased nutritional needs owing to heightened growth. Failure to meet these demands can result in both physical and cognitive health outcomes being compromised [5].

As this stage of development is crucial, a balanced diet with a focus on nutrient-dense foods is critical to support adequate growth, cognitive performance, and long-term health in adolescents. Promoting the consumption pattern and eating righteously makes a difference between childhood diseases and chronic adult diseases.

Nutritional assessment in adolescents

Assessing the baseline nutritional status of adolescents involves examining various biomarkers, including serum albumin, serum hemoglobin, and thyroid-stimulating hormone (TSH) levels [7]. Furthermore, nutritional status in adolescents can often be assessed through anthropometric measurements such as height, weight, waist circumference (WC) and hip circumference (HC), triceps and subscapular skinfold thickness, etc., and consequently, body mass index (BMI), waist-to-height ratio (WTHR), and waist-to-hip ratio (WHR) can be calculated [1]. Among these, BMI, as a measure to assess nutritional status, has been used for a long time and is very reliable, since it does not take into account age or sex [8]. Similarly, WTHR, as a tool for assessment (introduced in the mid-1990s), is not only a measure of body fat but also of its distribution, making it also an important tool in anthropometry [1].

Cognitive development and influencing factors in the modern era

Fast-moving technological and social factors are posing huge challenges to people and especially to younger generations, who have to adapt at lightning speed. It is largely the cognitive capacities, such as information processing, knowledge application, and decision-making skills [9], that dictate the ability to navigate such minefields. Cognition, in its broadest sense, refers to the mental processes that relate to acquiring knowledge and comprehension (beyond the collection of information) and include such concepts as reasoning, problem solving, and the ability to adapt in the face of new information or changing environments [10]. These cognitive skills are especially important in adolescence, a key stage of development, helping individuals navigate increasing complexities in academic, social, and environmental requirements [11].

The combination of social, psychological, and physiological factors influences the development, maintenance, and functioning of cognitive abilities. Nutrition, physical activity, and sleep are crucial components in a stimulating environment that fosters cognitive development [12]. Of these, nutritional status has a particularly vital role in improving cognitive performance, particularly in learning environments such as school and higher education environments. Adequate nutrition is believed to enhance brain function and facilitate learning, thus promoting better academic achievement [13].

Knowledge of how these factors interact is essential to promoting cognitive development during adolescence. Focusing on cognitive development determinants, especially through specific interventions aimed at fostering better nutrition and healthier behaviors, will improve both individual and social outcomes in a world where less and less is fixed.

Nutritional influence on cognitive abilities

Protein-calorie malnutrition has also been shown to impair learning, behavior, and overall organ health, further emphasizing the importance of proper nutrition [14]. In modern nutritional assessments, micronutrient deficiencies are now recognized as an important factor. Individuals with higher levels of B vitamins, as well as vitamins C, D, and E, tend to perform better on cognitive tests compared to those with deficiencies [15].

**Figure 1:**
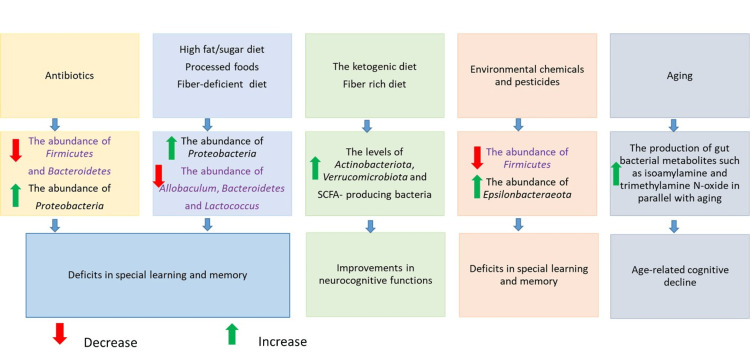
Association between various factors (nutritional interventions, age, antibiotics, and environmental factors such as chemicals), changes in the composition of the gut microbiome, and cognitive performance Reproduced from [16], under the terms of the Creative Commons Attribution License (CC BY 4.0). SCFA: short-chain fatty acid

Furthermore, microbiome-derived metabolites are also capable of modulating host metabolism, brain function, and cognitive performance by acting as epigenetic regulators [16]. Figure 1 shows the impact of various factors on cognitive performance through gut microbiome modifications.

Cognitive assessments

A comprehensive cognitive assessment aims to evaluate memory, reasoning, problem-solving, and intelligence. These evaluations use a variety of standardized tests, each of which seeks to assess particular cognitive functions. Among them are the Kaufman Test of Educational Achievement, the Wide Range Achievement Test 3, the Wechsler Intelligence Scale for Children and Adults, the Stanford-Binet Intelligence Scales, and Raven's Progressive Matrices (RPM) [1]. RPM, created in the 1930s, is a nonverbal and 'culture-free' test designed to examine the genetic and environmental influences on general intelligence [17]. Over time, Raven tests have become a widely recognized psychometric tool for assessing general intelligence across clinical, educational, and professional settings, offering a domain-independent evaluation of fluid reasoning through a single overall score [18]. Furthermore, the Wechsler Intelligence Scale for Children-Fifth Edition (WISC-V) is a standardized clinical tool designed to assess general cognitive abilities in children aged between six and 16 years and 11 months [19]. It evaluates problem-solving and reasoning skills across verbal, nonverbal, and visual domains, along with working memory and processing speed. Administered individually by a trained clinician, the test typically takes 60-90 minutes to complete. Scores are derived based on age-referenced norms from a national sample, providing standard scores for specific cognitive indices as well as broader measures of cognitive ability, including the Full-Scale Intelligence Quotient (Full-Scale IQ) [20].

Furthermore, neurocognitive function is examined through a computerized test battery, the Behavioural Assessment and Research System tests (BARS) [21]. The test battery included five tests to measure neurocognitive performance across various domains, such as Simple Reaction Time for response speed, Match to Sample for visual memory, Continuous Performance Test for sustained visual attention, Symbol Digit Test for information processing speed, and the Digit Span Test for assessing both attention and memory (Table 1).

**Table 1:** BARS measures and their cognition domain Reproduced from [21], under the terms of the Creative Commons Attribution License (CC BY 4.0). BARS: Behavioural Assessment and Research System tests

BARS Measures	Domain of Cognition Measured
Match to sample	Visual memory
Digit span	Short-term memory, attention
Continuous performance	Sustained attention
Finger tapping	Motor coordination
Simple reaction time	Response speed
Symbol digit	Information processing speed

## Review

Adolescence and nutrition

Adolescence is a developmental stage that starts with puberty and transitions into early adulthood. It is typically divided into three phases: early adolescence (ages 10-14), late adolescence (ages 15-19), and young adulthood (ages 20-24) [22]. This stage is distinguished by significant physical and sexual maturation, the pursuit of social and economic independence, the formation of personal identity, and the development of skills essential for adult relationships and roles, as well as the ability to outline thought. Adolescence also involves a rapid rate of growth, second only to that experienced in infancy [23].

Global demographics of adolescents and nutritional importance

Adolescents represent about 20% of the global population, with an even higher proportion in developing nations; for example, they account for around 26% of the population in El Salvador compared to 14% in the United States [24]. In 1995, approximately 914 million adolescents lived in developing countries, making up 85% of the global adolescent population, a figure projected to reach 1.13 billion by 2025 [24]. Adolescence is a period of rapid growth; proper nutrition is essential for reaching full growth potential [25]. Inadequate nutrition during this time can result in delayed or stunted linear growth and hinder organ development [26].

Nutritional deficiencies and concerns

According to the findings from the Global Burden of Disease Study 2021, it is estimated that approximately one in four people worldwide has anemia, affecting a total of two billion individuals. Nearly one in three women and around 40% of all children are impacted by this condition [27]. Although undernutrition, including stunting and wasting, has declined among children under five, there is an increasing prevalence of overweight and obesity in both children and adolescents [28]. Childhood obesity is linked to numerous immediate and long-term health risks, such as elevated cholesterol, triglycerides, and blood glucose, as well as type 2 diabetes, high blood pressure, and an increased likelihood of obesity and related health issues in adulthood [29].

Adolescent nutrition and long-term health outcomes

Adolescence is a critical life stage characterized by rapid physical, cognitive, and social development, with far-reaching implications for lifelong health and the well-being of future generations [30]. During this period, adolescents navigate significant changes in food environments, resulting in persistent challenges such as micronutrient deficiencies, food insecurity, and rising rates of overweight and obesity. Despite the clear importance of this life stage, adolescent nutrition remains underrepresented in policy and research frameworks, as funding and strategic initiatives often prioritize other age groups [31].

Optimal adolescent development is anchored in adequate nutrition, regular physical activity, and sufficient nutrient intake, which collectively establish the foundation for adult health and productivity [32]. Strengthening adolescent nutrition can break the intergenerational cycle of malnutrition, chronic disease, and poverty, leading to broad public health and socioeconomic benefits [33]. However, many adolescents worldwide continue to face overlapping health burdens, including undernutrition, poor reproductive health, violence, and growing exposure to non-communicable diseases [34]. Recognizing these challenges, global initiatives such as the Lancet Commission on Adolescent Health and Well-being have emerged to mobilize evidence-based action, foster collaboration among stakeholders, and prioritize adolescent health within broader development agendas [33].

Nutritional assessment of adolescents

Nutrition plays an essential role in supporting growth and development from infancy through adolescence, with nutrient demands increasing during the teenage years [35]. Schools offer a valuable opportunity to reach not only adolescents but also school staff, families, and the wider community, facilitating large-scale nutrition interventions. During adolescence, as in other life stages, anthropometric measurements are useful for assessing nutritional status.

Adolescence presents a critical window for establishing a foundation for lifelong health through proper nutrition [32]. Nutritional deficiencies during this stage can have extensive impacts, particularly for adolescent girls, who, if malnourished, are at greater risk of bearing undernourished children and transmitting undernutrition in future generations [31]. In developing countries, children with mild to moderate malnutrition often survive into adolescence, where their malnutrition becomes chronic and typically only detectable through anthropometric assessments [36]. Conversely, adolescents who were well-nourished in childhood may develop malnutrition during their young years due to poor dietary habits, sometimes driven by societal pressures to achieve thinness [3]. Many developing nations now face a dual burden of undernutrition and rising overnutrition rates among adolescents [37]. Traditionally, adolescents have received limited attention in nutrition assessments, partly due to their relatively low mortality compared to other age groups, resulting in a lower prioritization of their nutritional needs [24].

Anthropometric assessments

Anthropometry is the most affordable, non-invasive, and universally applicable method for evaluating body composition, size, and proportion [38]. It is easy to perform in different epidemiological or clinical settings. It aims to collect measurements of the human body at a total body and/or regional level using simple devices (stadiometer, weight scale, meter, gauges, compasses, skinfold calliper, etc.).

Application in Nutritional Status Assessment

Anthropometric parameters are useful for analysing the relationships between lifestyle (eating habits and level of physical activity) and nutritional status because, although linked to genetic heritage, they are also influenced by lifestyle, eating habits, physical activity, and environmental, social, and cultural factors [38].

BMI

While BMI is widely adopted to screen for assessing nutritional status in adolescents, its limitations, the inability to distinguish adipose tissue from lean muscle mass, and the inability to account for ethnic variations in body composition must be recognized [39]. The sensitivity and specificity of BMI as a nutritional indicator can be limited [40]. Age is another factor that can affect the functional significance of BMI, as adults tend to lose fat-free mass and gain fat mass with age [41]. Additionally, edema can influence the accuracy of BMI, such as severely undernourished adults may develop edema, which artificially raises their weight, causing their BMI to appear more normal than it truly is [42]. Furthermore, the universal BMI cut-off may not be suitable for all populations, restricting its effectiveness as a precise screening tool for assessing undernutrition in adults [43]. Considering this, the World Health Organization (WHO) has proposed ethnicity-specific BMI thresholds of ≥23 kg/m² for overweight and ≥25 kg/m² for obesity [44] while <18.5 kg/m² for underweight and 18.5-22.9 kg/m² for normal range in Asian populations [45].

Sri Lankan studies indicate that the standard WHO BMI cut-off values may underestimate the obesity risk in Asian populations [44]. Therefore, incorporating complementary anthropometric measures, such as the WTHR, may improve the accuracy of nutritional assessments [45]. Although direct body composition assessment methods, such as dual-energy X-ray absorptiometry (DXA) and magnetic resonance imaging (MRI), provide higher precision, their application in epidemiological studies is limited due to high costs and logistical issues [46].

Alternative Anthropometric Measurements

Several direct anthropometric measurements are available, including WC, HC, WHR, bioelectrical impedance, hydro densitometry (underwater weighing), isotope dilution, sagittal abdominal diameter, DXA, MRI, computed tomography (CT) scans, and skinfold thickness [42]. These methods are considered more accurate for assessing visceral fat accumulation, metabolic profiles, and disease risks, as they provide precise body fat measurements. However, direct methods often require significant time and financial investment, advanced facilities, highly trained personnel, and complex procedures and are limited by the lack of retrospective data and technical challenges such as sport-specific technical skills associated with differences in biological maturity status [46].

Biochemical assessments

Information obtained from performing the various biochemical tests must be interpreted and evaluated in terms of significance and compared with normal standards developed for appropriate sex and age groups. Evaluating biomarkers and analysing nutrient intake from food and dietary supplements are the two primary methods for assessing the nutritional status of a population [47]. Furthermore, a range of laboratory parameters commonly used in clinical practice, such as complete blood count, lipid profile, electrolytes, and liver function tests, can offer valuable information about the nutritional status of a person [48]. These tests can help identify nutrient deficiencies, determine the underlying causes of malnutrition, monitor the progress of nutritional therapy, assess disease severity and activity, and track changes in body composition. For chronically malnourished patients, laboratory results are useful in detecting deficiencies in vitamins (such as C, D, E, K, thiamine, B6, B12, and folic acid) and trace elements (such as zinc, selenium, and iron) while also aiding in the management of supplementation therapies [49]. Because during adolescence, iron requirements rise due to increased body size, growth demands, and, in girls, the onset of menstruation. Additionally, sufficient calcium and vitamin D are essential for maintaining optimal bone health [50]. 

Protein and Inflammation Markers

Serum albumin, a key indicator of protein status, reflects long-term protein intake and helps identify cases of protein malnutrition. Albumin, the most abundant protein in plasma, plays an important role in regulating body fluid distribution, maintaining acid-base balance, and binding essential components in the bloodstream [51]. C-reactive protein (CRP), produced by hepatocytes, is widely used as a marker of inflammation. During inflammatory conditions, CRP levels typically increase, whereas albumin concentrations tend to decrease, reflecting the acute-phase response [52].

Hemoglobin and Anemia

Iron status should be evaluated using multiple markers that reflect different aspects of iron adequacy [53]. Hemoglobin levels are such an important measure, with low levels indicating anemia, often caused by iron deficiency, which is a common issue in adolescents that can affect physical and cognitive development [54]. Anemia is a condition characterized by an insufficient number of red blood cells or a reduced ability of these cells to carry oxygen, failing to meet the body's physiological needs [55]. Depression, irritability, loss of appetite, apathy, evidence of impaired learning and cognition, and slowed growth rates have all been associated with iron deficiency anemia. Accurate characterization of anaemia in adolescents is essential for understanding its prevalence and distribution, guiding public health interventions, and ensuring comprehensive and integrated health services. This includes promotive, protective, preventive, curative, rehabilitative, and palliative care, with a focus on primary care and essential public health functions throughout adolescence and beyond [56]. Moreover, the WHO recommended iron and folic acid supplementation as a preventive measure against anaemia in adolescents [57]. The emphasis on adolescents highlights their higher likelihood of poor dietary habits, the increased risk of post-pubertal anaemia in girls, and their susceptibility to infections. The focus on adolescents underscores their greater tendency toward poor dietary habits, the heightened risk of post-pubertal anaemia in girls, and their increased vulnerability to infections [57].

Micronutrient Biomarkers

Blood tests for micronutrients, including vitamins A, D, and B complex, as well as minerals like iron, zinc, and iodine, are essential for detecting deficiencies during periods of rapid growth [58]. Sufficient intake of vitamins and minerals, including iron, zinc, copper, selenium, vitamin B12, and folate, is crucial for maintaining optimal health. The concentration of zinc in serum or plasma is currently the only biochemical indicator recommended for evaluating the zinc status of populations [59]. Vitamin K status is evaluated by assessing blood clotting rates, while vitamin C levels are typically determined through serum or plasma concentrations [53].

Serum vitamin B12 is a biomarker of vitamin B12 deficiency, and the metabolites, methylmalonic acid (MMA) and homocysteine, are functional indicators [60]. Assessment of copper status has challenges like those of other minerals, and serum copper is used widely as a biomarker of copper status. Biochemical biomarkers of vitamin A status are serum retinol, serum retinol-binding protein (RBP), breast milk retinol, dose-response tests, isotope dilution methodology, and serum retinyl esters [61]. Vitamin A deficiency often persists into adolescence. For example, in Southeast Asia, 23% of children aged between five and 15 years were found to have serum retinol concentrations below 0.7 μmol/L, although inflammation markers were not assessed, and nearly 3% exhibited non-blinding mild xerophthalmia [62]. Selenium status is determined using serum, plasma, or red blood cell selenium levels. Chromium and manganese levels can be measured in different blood fractions. Iodine status is generally assessed through urinary iodine concentration, which serves as a reliable indicator of recent iodine intake [53].

Thyroid Function

TSH levels are also assessed to evaluate thyroid function, which is essential for growth and metabolic regulation [63]. Hypothyroidism, a condition marked by decreased thyroid hormone levels, is linked to hypometabolism. This state is characterized by lower resting energy expenditure, weight gain, elevated cholesterol levels, decreased fat breakdown (lipolysis), and reduced gluconeogenesis [64].

Urinary Analysis and Creatinine Measures

Urine analysis can provide information on micronutrient status. Creatine is metabolized into creatinine at a relatively stable rate, serving as an indicator of muscle mass. Creatinine excretion is closely linked to lean body mass and body weight. The creatinine height index (CHI) is a measure of lean body mass and is calculated using the formula: CHI (%) = (measured 24-hour urinary creatinine × 100) ÷ normal 24-hour urinary creatinine. However, urinary creatinine excretion can be influenced by factors such as renal insufficiency, meat consumption, physical activity, fever, infections, and trauma. Additionally, collecting 24-hour urine samples poses practical challenges, further limiting the method's routine application [64].

Dietary assessments

Dietary habits are closely linked to the academic performance of adolescents. For example, frequent consumption of fast food is associated with poorer academic outcomes. These foods are typically low in nutritional quality and fail to meet established nutrient guidelines. More than 50% of fast-food meals exceed sodium recommendations, while fewer than 25% comply with trans-fat guidelines. Additionally, less than one-third of these meals provide sufficient calcium and iron, and fewer than 20% meet vitamin A requirements [65]. Insufficient intake of essential nutrients, like iron, combined with high levels of fat and added sugar from fast food, has been shown to negatively impact school performance and contribute to metabolic disorders such as insulin resistance and obesity [65].

Skipping breakfast is another dietary habit that can adversely affect adolescents. Studies have shown that children exhibit improved spatial and short-term memory after consuming breakfast, likely due to the stabilization of blood glucose levels following a meal [66]. The importance of breakfast for cognitive function and learning is well-documented, as it provides a steady release of energy and supports the intake of essential micronutrients like iron, iodine, and vitamin A, regardless of behavioural or other influencing factors [67].

Diet is especially important during adolescence, a period of high brain metabolic demands, as brain glucose consumption remains elevated until the ages of 16 to 18 years. Furthermore, the nutritional components of individual foods and the overall quality of a person’s diet, as indicated by a high diet quality index or healthy eating patterns, are linked to better academic performance [68]. This connection may also reflect underlying socioeconomic status and personal characteristics, which influence both diet quality and academic achievement.

Methods for Assessing Dietary Intake

There are several tools used to assess the dietary intake of individuals, such as 24-hour dietary recall, food frequency questionnaires (FFQs), dietary histories, and food diaries.

The 24-hour dietary recall is a widely used method to assess the dietary intake of an individual by asking them to recall all foods and beverages consumed over the previous 24 hours [69]. This method provides a detailed description of recent dietary habits, including portion sizes, meal timing, and preparation methods, enabling the estimation of energy and nutrient intake. It is especially valuable in identifying specific nutrient deficiencies or excesses within a short timeframe [70]. However, its accuracy depends on the participant's memory and honesty, and it may not reflect typical dietary patterns if the day recalled was unusual for the individual. Despite these limitations, it is a cost-effective and practical tool for large-scale dietary studies and clinical assessments [71].

FFQs are designed to evaluate long-term dietary patterns by recording how often certain food groups are consumed over a specific period, such as weeks or months. FFQs categorize foods into groups (e.g., fruits, vegetables, dairy) and assess the frequency and portion sizes of their consumption [72]. This method offers insights into dietary quality, diversity, and potential nutrient inadequacies or excesses. While FFQs are less detailed than 24-hour recalls, they are useful for capturing habitual dietary patterns and are often used in epidemiological studies to explore the relationship between diet and health outcomes [73]. However, their reliance on self-reported data can introduce biases and require careful design and validation to ensure reliability [74].

**Figure 2:**
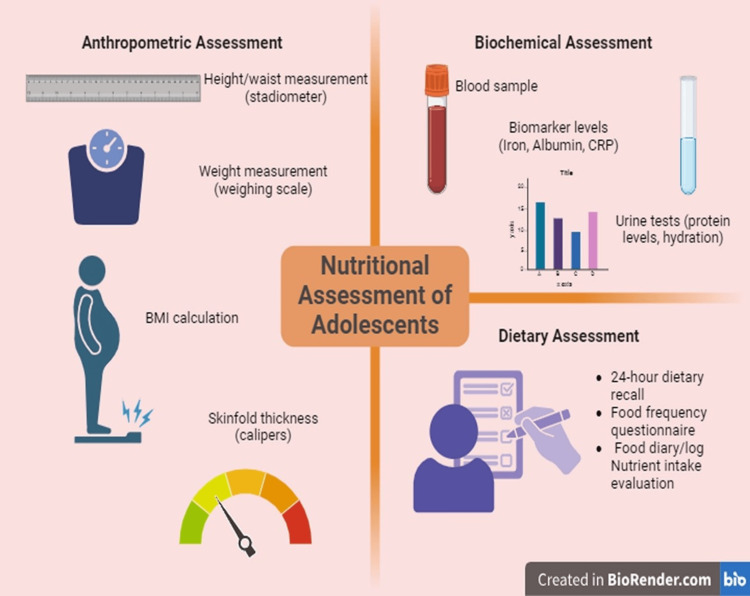
Overall summary of nutritional assessment of adolescents Created by the authors with BioRender.com.

Dietary history and food diaries provide a detailed view of the dietary habits of an individual over several days or weeks. Dietary history involves an in-depth interview to assess usual food intake, meal patterns, and dietary preferences, often capturing variations across weekdays and weekends [75]. On the other hand, food diaries require participants to record all food and drink consumed over a defined period, including quantities, preparation methods, and even accompanying emotions or contexts [76]. These tools are valuable for identifying patterns, such as binge eating or skipping meals, and for calculating precise nutrient intake. Although they offer detailed and personalized dietary details, food diaries can be time-intensive for both participants and researchers and may lead to altered eating behaviours due to the act of recording [77]. Figure 2 shows the overall summary of the nutritional assessment of adolescents in terms of anthropometric, biochemical, and dietary assessment.

Academic performance and cognitive function in adolescents

Academic performance refers to the achievements of students in education or learning. With increasing competition in the job market, the importance of excelling academically has gained significant attention from parents, policymakers, and educational authorities. Academic achievement is an important aspect of child development, as skills in areas such as reading and mathematics significantly influence various life outcomes [78]. It includes educational attainment, job performance, income levels, physical and mental health, and overall life expectancy [79]. Parents often emphasize academic success and that strong academic results will lead to better career opportunities and job stability [80]. In addition, academic performance is frequently used as a criterion in interviews and recruitment processes, highlighting its important role in shaping career prospects for adolescents [81].

Factors influencing cognitive function and academic performance

A variety of factors influence cognitive function and academic performance. Psychosocial and physiological elements significantly impact the development, functioning, and maintenance of cognitive abilities, affecting academic outcomes [51]. Key contributors to cognitive development include proper nutrition, regular exercise, and adequate sleep.

Several research studies on academic achievement have focused on identifying key factors that influence academic development and how these can be integrated into interventions and instructional strategies to enhance performance and address learning difficulties [82]. These studies investigate various determinants, including individual, environmental, and institutional factors, to understand how they collectively impact learning outcomes [83]. Individual factors often include cognitive abilities, emotional intelligence, motivation, and self-regulation, all of which play crucial roles in shaping academic performance [78,79].

Key categories influencing academic success

There are two primary categories of factors essential for academic success [80]. The first category involves foundational domain-specific skills. For reading and comprehension, meta-linguistic abilities such as phonological processing, orthographic knowledge, morphological awareness, and fluency and comprehension strategies are essential. Similarly, in mathematics, skills like number sense and fact retrieval form a fundamental basis for learning [80].

The second category includes cognitive abilities, including working memory, reasoning, and executive function. Working memory involves the simultaneous storage and manipulation of information. Reasoning is the ability to solve novel and complex problems [84]. Executive function includes cognitive and socio-emotional processes that support goal-directed behaviour, such as flexible thinking, self-regulation, and self-control. Together, these domain-specific skills and cognitive abilities are important for improving academic growth and addressing challenges in learning [80].

Cognitive theories and academic achievement 

The relationship between cognitive abilities and academic achievement is explained well through two major cognitive theories: investment theory and dual-process theory [85]. Investment theory suggests that the development of cognitive abilities is created by biological, genetic, and health factors rather than education. According to this theory, academic performance stems from the application of cognitive abilities combined with environmental stimulation, such as the opportunities provided by educational settings. Cognitive abilities are considered the foundation upon which academic achievement is built [80].

Similarly, the dual-process theory of higher cognition suggests that individuals process familiar information automatically, requiring minimal cognitive resources, while novel information is processed in a controlled and resource-intensive manner [86]. This theory implies that the role of cognitive abilities in academic tasks depends on how efficiently the task can be performed, which is closely attached to the long-term memory of task-related knowledge [87]. During the early stages of learning, academic tasks demand significant cognitive effort. However, as knowledge accumulates and learning progresses, dependence on cognitive abilities decreases, and individuals begin to draw directly from long-term memory to perform tasks more efficiently [80].

Nutrition of adolescents in Sri Lanka

Adolescents make up approximately 17% of the Sri Lankan population. Despite the country experiencing no significant food shortages and offering extensive free maternal and child health services, malnutrition continues to affect nearly one-fifth of children under five and many women, presenting a striking paradox [88]. Adolescence is a period of rapid physical, cognitive, social, and emotional changes, significantly influencing eating habits and overall health. The accelerated growth and development during this stage lead to heightened energy and nutrient demands. However, numerous psychosocial factors, such as newfound independence, peer influence, the search for self-identity, sociability, concerns about appearance, media exposure, food availability, and economic status, can affect food choices and eating behaviours. When these factors conflict with nutritional needs, health can be adversely impacted [89].

Nutritional status of Sri Lankan adolescents

A 2005 study conducted by the Medical Research Institute found that among school children aged between 10 and 15 years, the prevalence of thinness, stunting, and overweight was 47.2%, 28.5%, and 2.2%, respectively [89]. The study also reported that 11.1% of these children were anemic, while 0.4% had vitamin A deficiency.

Rapid changes in diets and lifestyles resulting from industrialization, urbanization, economic development, and market globalization have accelerated during the last decade [90]. This has had a significant impact on the health and nutritional status of the population. The impact is notable in developing countries and countries in transition like Sri Lanka [91]. While such changes have resulted in improved standards of living and greater access to services, there have also been significant negative consequences due to inappropriate dietary patterns, decreased physical activities, and increased tobacco use, resulting in a corresponding increase in diet-related chronic diseases [92].

Micronutrient deficiencies and emerging concerns

According to the national nutrition and micronutrient survey among school adolescents aged between 10 and 18 years in Sri Lanka in 2017 [89], several nutritional issues have been identified among schoolchildren aged between 10 and 18 years in Sri Lanka based on the WHO cut-off values for public health significance. Thinness, or underweight, had a high prevalence among schoolchildren in Sri Lanka, observed nationally at 26.9% and at the provincial level between 23.3% and 22.5%. Overweight and obesity had a low national prevalence of 9.7% and ranged between 5.6% and 16.3% in seven out of nine provinces, with moderate levels in the Western Province (16.3%) and North Central Province (11.9%). Stunting remained a low-prevalence issue nationally (13.7%) and across provinces (5.8%-17.8%). Anaemia presented as a mild public health concern both nationally (8.8%) and provincially (4.3%-15.7%).

Iron deficiency was a moderate concern nationally (22.1%) and in six provinces (16.1%-29.9%), while anaemia related to iron deficiency was no longer a significant national issue (3.8%) but persisted mildly in the Northern Province (7.3%). Vitamin A deficiency was no longer a problem nationally (0.1%) or provincially (0.0-0.4%), whereas vitamin D deficiency was emerging as a significant issue nationally (13.2%) and provincially (0.7%-32.2%). Iodine levels were optimal nationally (137.9 μg/dL) and in most provinces (133.1-175.8 μg/dL), though insufficient levels were reported in the Western (94.2 μg/dL) and Uva (89.0 μg/dL) provinces. Zinc deficiency remained a significant public health concern both nationally (29.4%) and provincially (17.6-46.1%).

In Sri Lanka, recent data on the nutritional status of school adolescents is lacking. According to available literature, there is a gap in recent information regarding the nutritional and health status of this age group. Because of the wide array of factors affecting nutritional status among school adolescents, conducting a study that identifies basic, underlying, and immediate causes of malnutrition is essential.

Relationship between nutritional status and cognitive function

The Role of Nutrition in Cognitive Development and Academic Performance

Nutrition plays an essential role in both cognitive development and academic performance [93]. It is widely recognized that nutritional status significantly influences academic achievement and cognitive abilities in school and higher education [94]. As a fundamental daily requirement for all living beings, nutrition has a significant impact on cognition. Defined as the intake of food to meet the dietary needs of the body, nutrition forms the foundation of good health [95]. However, in the fast food-driven adolescent generation today, convenience often takes priority over nutritional value, with exotic burgers readily consumed while vegetables are often overlooked, reflecting a dramatic shift in dietary habits [96].

Components of Nutritional Assessment

Nutrition has two primary aspects: the intake of food, which focuses on calories and nutrient content, and the outcomes of this intake, as evidenced by physical indications [3]. Nutritional assessment thus requires both dietary pattern analysis and anthropometric measurements [97]. Dietary intake is an important component of nutritional assessment that is influenced by both the quantity and quality of food consumed [98]. Various methods, such as single or multiple 24-hour dietary recalls, weighed diet records, self-reported diet histories, and FFQ, can be used to evaluate dietary patterns. Among these, the 24-hour recall is one of the most widely employed techniques [99].

Physical measurements of nutrition are assessed through anthropometric measurements, including height, weight, waist and hip circumference, and skinfold thickness at sites like the triceps and sub-scapular region [100]. These measurements are used to calculate indices such as BMI, WTHR, and WHR. BMI has long been a reliable measure of nutritional status, as it is unaffected by age or sex. More recently, WTHR has emerged as an important tool, not only for assessing body fat but also its distribution, further enhancing its value in nutritional and anthropometric studies [49].

Impact of Nutrition on Cognitive Development

The importance of proper nutrition in cognitive development, which in turn impacts educational achievements [101]. Furthermore, research findings indicate that nutrition affects thinking skills, behavior, and overall health, all of which influence academic performance [102]. While most of this research has focused on adults, there is a notable lack of literature exploring the relationship between nutrition and cognitive function in children and adolescents.

Inadequate nutritional status can adversely affect the growth and development of children [103]. Children who experience poor nutrition tend to have smaller brain sizes, with brain cells that are 15%-20% smaller compared to those of well-nourished children [66]. Among adolescents, nutritional deficiencies can hinder mental growth and development, leading to delays in cognitive progress and increased absences from school due to illness [104]. The long-term consequences of poor nutrition during childhood extend into adulthood, impacting brain development, intellectual quotient, and scholastic achievement [67].

Proper brain function is essential for effective cognition and the ability to perform organized behaviours. The continuous activity of the brain is essential for the survival of an organism, as it facilitates the ongoing execution of both essential voluntary and involuntary functions [105].

**Figure 3:**
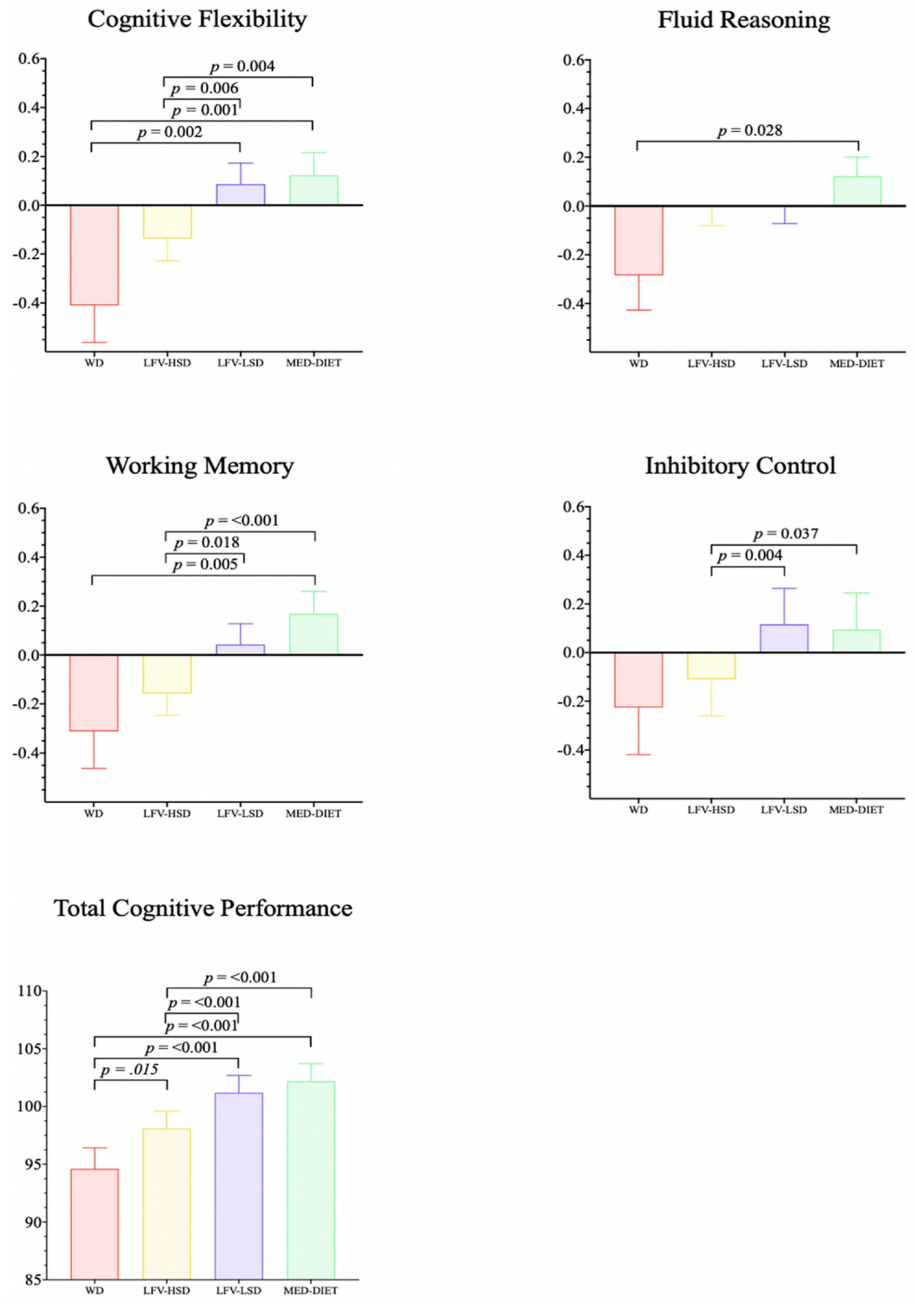
Comparisons between clusters according to cognitive domains WD: Western diet; LFV–HSD: low fruit and vegetable, high–sugar diet; LFV–LSD: low fruit and vegetable, low–sugar diet; MedDiet: Mediterranean diet Reproduced from [106], under the terms of the Creative Commons Attribution License (CC BY 4.0).

**Figure 4:**
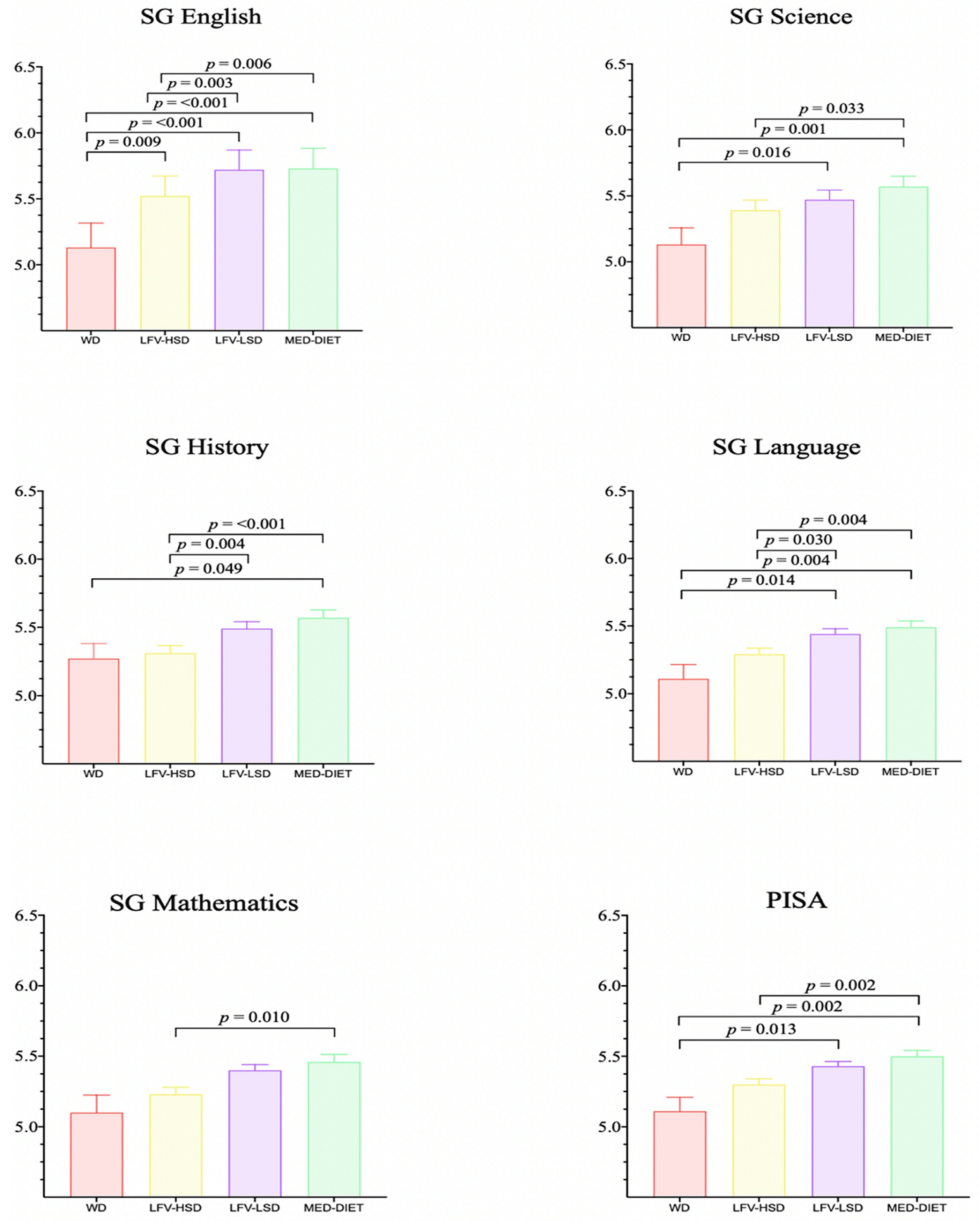
Results of the mixed models for academic achievement WD: Western diet; LFV–HSD: low fruit and vegetable, high–sugar diet; LFV–LSD: low fruit and vegetable, low–sugar diet; MedDiet: Mediterranean diet; SG: school grades Reproduced from [106], under the terms of the Creative Commons Attribution License (CC BY 4.0).

Figure 3 shows the significant differences between the four clusters: the Mediterranean diet (MedDiet), the Western diet (WD), the low fruit and vegetable, high-sugar diet (LFV-HSD), and the low fruit and vegetable, low-sugar diet (LFV-LSD)-according to the cognitive domains: working memory, cognitive flexibility, inhibitory control, fluid reasoning, and total cognitive performance [106]. Furthermore, they found that there were significant differences between the above four clusters in academic achievement: language, English, mathematics, science, history, and the Programme for International Student Assessment (PISA) (Figure 4).

Nutritional Requirements for Cognitive and Physical Growth

Balanced nutrition is important for school-age children, a stage characterized by rapid growth, heightened activity levels, and the development of both physical and cognitive functions [107]. The quality of food and proper nutrition are closely linked to brain development and cognitive performance, both of which are vital for the overall health and well-being of children [76]. From a neuropsychological perspective, adequate nutrition is fundamental for maintaining healthy brain function, supporting optimal learning, and enhancing academic achievement [108].

Dietary Patterns and Cognitive Function

Diets rich in omega-3 fatty acids and antioxidants, such as the MedDiet, are consistently associated with better cognitive performance in adolescents [106]. However, such findings may be confounded by socioeconomic factors, as families adhering to the MedDiet often have higher education levels and access to healthcare [6]. In contrast, high-sugar diets (e.g., LFV-HSD) showed associations with poorer academic outcomes, though causality remains unconfirmed due to the majority of these studies being associated with self-reported dietary data [109]. Notably, Sri Lankan studies on diet-cognition links are limited, questioning about cultural adaptability of global evidence (e.g., rice-based diets vs. MedDiet). In recent years, research has shifted from examining the effects of isolated nutrients on brain health to exploring the impact of overall dietary behaviours or patterns, such as consuming junk food or adhering to a MedDiet rich in fruits, vegetables, and fish [110]. According to the findings of [106], higher adherence to Mediterranean-style-based patterns and better food quality choices are associated with improved cognitive and academic achievements. Table 2 reports the assessment of cognitive performance tasks and academic achievement results by [106].

**Table 2:** Cognitive and academic measurement (n = 1296) Data are expressed as mean ± standard deviation or count (percentage). The significant p-values are shown in bold. Student’s t-test for independent samples. Academic achievement Chilean scale up to 7.0 score; Academic- Programme for International Student Assessment (PISA) average: language, mathematics, and science average. (y): years; (p): percentile; (s): score; BMIz: Body Mass Index z-score; WD: Western diet; LFV–HSD: low fruit and vegetable, high–sugar diet; LFV–LSD: low fruit and vegetable, low–sugar diet; MedDiet: Mediterranean diet Reproduced from [106], under the terms of the Creative Commons Attribution License (CC BY 4.0).

Variables	All	WD	LFV-HSD	LFV-LSD	MedDiet
	(n = 1296)	(n = 56)	(n = 365)	(n = 547)	(n = 328)
Cognitive tasks					
Cognitive flexibility					
Trail-making test A (p)	100.0 ± 14.7	91.4 ± 13.5	97.7 ± 14.5	101.1 ± 14.3	102.2 ± 14.9
Trail-making test B (p)	100.0 ± 14.7	93.3 ± 15.3	97.1 ± 14.1	101.5 ± 14.6	101.8 ± 14.7
Digit coding symbol (p)	100.0 ± 14.7	92.3 ± 13.4	97.2 ± 14.6	101.6 ± 14.5	101.8 ± 14.4
Working memory					
Memory forward (p)	100.0 ± 14.4	92.0 ± 14.9	97.2 ± 13.6	101.4 ± 14.2	102.1 ± 14.4
Memory reverse (p)	100.0 ± 14.4	94.8 ± 14.6	96.7 ± 14.3	101.2 ± 13.9	102.4 ± 14.2
Inhibitory control					
Go/No-Go (p)	100.0 ± 14.7	93.9 ± 15.4	98.4 ± 15.5	100.5 ± 14.6	101.9 ± 13.4
Fluid reasoning					
Problem-solving (p)	100.0 ± 14.5	92.8 ± 13.4	97.4 ± 13.9	101.3 ± 14.3	102.1 ± 14.8
Progressive matrices (p)	100.0 ± 14.3	94.1 ± 11.4	97.6 ± 13.4	101.6 ± 14.6	101.3 ± 14.5
Academic achievement					
English (s)	5.62 ± 0.9	5.16 ± 0.9	5.52 ± 0.9	5.68 ± 0.8	5.72 ± 0.9
History (s)	5.45 ± 0.8	5.20 ± 0.6	5.31 ± 0.8	5.50 ± 0.8	5.56 ± 0.8
Language (s)	5.40 ± 0.8	5.09 ± 0.7	5.31 ± 0.8	5.45 ± 0.8	5.47 ± 0.8
Mathematics (s)	5.35 ± 1.0	5.09 ± 0.9	5.24 ± 1.0	5.42 ± 0.9	5.42 ± 1.0
Science (s)	5.45 ± 0.8	5.10 ± 0.8	5.39 ± 0.9	5.46 ± 0.8	5.54 ± 0.8
Academic-PISA average	5.40 ± 0.8	5.09 ± 0.7	5.31 ± 0.8	5.44 ± 0.7	5.47 ± 0.8

**Figure 5:**
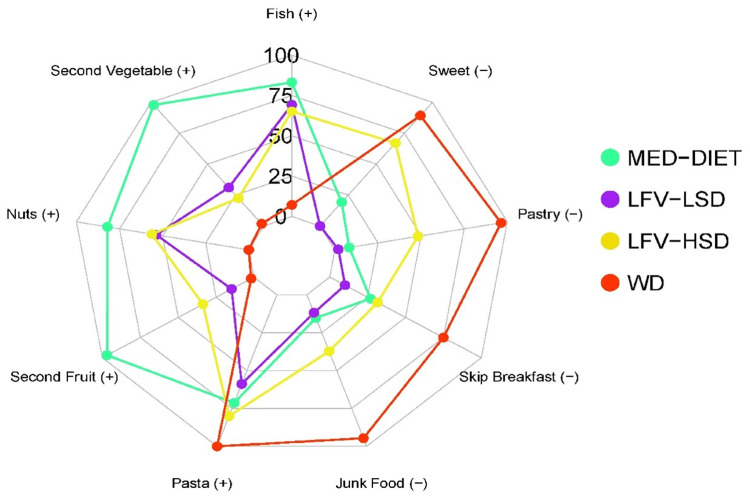
Radar plot of the percentage of adherence to each indicator used in the analysis according to each cluster Reproduced from [106], under the terms of the Creative Commons Attribution License (CC BY 4.0). WD: Western diet; LFV–HSD: low fruit and vegetable, high–sugar diet; LFV–LSD: low fruit and vegetable, low–sugar diet; MED-DIET: Mediterranean diet

Furthermore, Figure 5 illustrates the radar plot analysis of the outcomes attributed to each cluster for the specified indicator. Given that the Mediterranean Diet Quality Index (KIDMED) indicators represent a dichotomous result, with the emphasis on the positive fulfillment of each of these indicators. According to the findings of this study, there were statistically significant differences in cognitive performance (cognitive flexibility p = 0.006; working memory p = 0.018; inhibitory control p = 0.004; total cognitive performance p ≤ 0.001) and academic achievement (English p = 0.003; History p = 0.004; Language p = 0.030) in favour of the LFV-LSD cluster (low sugar consumption) [106]. Therefore, it is important to investigate the potential influence of diet on learning among young people. However, few studies have explored the relationship between cognitive function and diet in Sri Lankan youth. 

## Conclusions

This review highlights the critical role of nutrition in adolescent cognitive development and academic performance, emphasizing that inadequate intake of protein, energy, and micronutrients like iron, zinc, and vitamin D negatively affects brain function and mental health. Nutrient-rich diets, such as the MediDiet, are associated with better outcomes. In Sri Lanka, adolescents face a dual burden of under- and overnutrition due to rapid lifestyle changes, necessitating school-based nutrition interventions and public health strategies. Future longitudinal studies are needed to establish causal links between diet and cognition, reduce nutritional inequalities, and break cycles of malnutrition and poor academic achievement. To address the dual burden of malnutrition, it is recommended that policymakers prioritize school-based programs such as mandatory nutrition education and micronutrient supplementation (e.g., iron, vitamin D) as required for adolescents. They should focus more on longitudinal research, including cohort studies tracking dietary patterns, biochemical markers, and academic performance.
